# Therapeutic Targets and Personalized Medicine in Cardiac Disease

**DOI:** 10.3390/jpm13111534

**Published:** 2023-10-26

**Authors:** Elizabeth Vafiadaki, Irene C. Turnbull, Despina Sanoudou

**Affiliations:** 1 Center of Basic Research, Biomedical Research Foundation of the Academy of Athens, 11527 Athens, Greece; dsanoudou@med.uoa.gr; 2 Cardiovascular Research Institute, Icahn School of Medicine at Mount Sinai, New York, NY 10029, USA; irene.turnbull@mssm.edu; 3 Clinical Genomics and Pharmacogenomics Unit, 4th Department of Internal Medicine, Attikon Hospital Medical School, National and Kapodistrian University of Athens, 11527 Athens, Greece; 4 Center for New Biotechnologies and Precision Medicine, Medical School, National and Kapodistrian University of Athens, 11517 Athens, Greece

## 1. Special Issue Overview

Despite extensive research that has achieved notable advancements over the last decades, cardiovascular disease (CVD) remains the leading cause of death worldwide, with millions affected around the world. This can be attributed to various factors, including considerable challenges in patient diagnosis and/or prognosis, limited efficacy of currently available therapeutic options that often alleviate symptoms rather than cure disease, and the highly variable response to drug treatment. The remarkable technological advancements and scientific breakthroughs accomplished during recent years have unveiled a horizon of new possibilities, the exploration of which could hold promise in the era of precision medicine. This Special Issue of the *Journal of Personalized Medicine* features a collection of articles focused on the latest scientific advances in CVD, specifically emphasizing therapeutic targets and the development of innovative approaches aiming towards effective personalized therapy. 

## 2. Therapeutic Targets

Among the various genes implicated in dilated and arrhythmogenic cardiomyopathies, accumulating evidence points to phospholamban (*PLN*) and desmoplakin (*DSP*) as crucial players [1]. Articles in this Special Issue report the latest research findings on specific *PLN* and *DSP* variants, unveiling key pathophysiological features and pointing to potentially promising approaches for personalized therapy. In particular, the pathophysiology of a *PLN* deletion mutation (c.40_42delAGA, p.Arg14del) was studied in detail following the generation of a humanized knock-in mouse model [2,3]. In-depth characterization of this mouse model identified an arrhythmogenic phenotype originating from the heart’s right ventricle (RV), presenting distinct electrocardiographic features similar to human patients. Studies on isolated cardiomyocytes revealed significant calcium defects and arrhythmogenic propensity present only in RV cells [2]. These pathological defects were observed in mice of young age, highlighting their importance as early aberrations contributing to disease progression and, at the same time, pointing towards the potential of targeted therapy in patients carrying this *PLN* genetic variant.

Regarding *DSP*, a mutational hot spot exists in the encoded protein’s amino-terminal region consisting of four arrhythmogenic cardiomyopathy-linked variants (S299R, S442F, R451G, and S507F). Unlike others, these four DSP variants cause decreased DSP levels via increased protein degradation triggered by hypersensitivity to calpain proteolysis. The study of Hoover et al. [4] explored the potential of preventing this aberration by occluding the variant-exposed calpain target site. This was achieved by introducing a secondary single-point mutation at amino acid position 518 (L518Y) that acted as a ‘molecular band-aid’ to protect from calpain cleavage [4]. Even though partial protection was achieved, most likely due to inherent issues associated with the size, position, and mobility of the secondary L518Y mutation, this proof-of-concept study highlights the promise of adopting such a therapeutic approach in future precision medicine strategies. 

In addition, the two comprehensive review articles of this Special Issue provide a critical overview of current knowledge on gamma-secretase and heat shock protein-90 (Hsp90), two molecules with emerging essential functions in the heart [5,6]. Although gamma-secretase has been extensively studied in the context of Alzheimer’s disease, its involvement in cardiac regulation has also become apparent. Specifically, it has been shown to be responsible for processing several substrates relevant to cardiac development and function [5]. Importantly, disruption of gamma-secretase activity is detrimental to cardiac function, highlighting its essential role in the heart. While the potentially beneficial effect of gamma-secretase modulators in cardiac disease treatment is currently unclear, findings from Alzheimer’s disease studies suggest the need for a precision medicine approach to avoid serious side effects [5]. Future development of modulators tailored to specific gamma-secretase pathways could provide targeted strategies in combating cardiac disease. A similar scheme could also be employed for Hsp90, a molecular chaperone regulating many proteins implicated in multiple and divergent cardiac pathways [6]. According to findings from Hsp90 inhibitor studies, a precision type of approach that modulates specific Hsp90 activity, rather than systemic inhibition, would be more beneficial for heart disease treatment [6]. 

## 3. Therapeutic Approaches

Apart from new therapeutic targets per se, improved therapeutic approaches are also being pursued. These include unraveling drug-nutrient-genome interactions (DNGIs), a relatively unexplored territory impacting the safety and efficacy of CVD drugs in different patients. Specifically, emerging evidence suggests that DNGIs represent a critical parameter affecting drug pharmacokinetics (e.g., drug absorption, distribution, metabolism, and excretion) and pharmacodynamics (drug action) [7]. Dietary elements such as food, beverages, and supplements have been shown to modulate both drug efficacy and safety. At the same time, different genetic variants can further complicate individual responses to various drugs. The cytochrome P50 (CYP) class of genes represents a classic example, with multiple genetic polymorphisms on different CYP genes being directly implicated in DNGIs [7]. With continuously increasing new knowledge, it is expected that future drug administration guided by genetically informed nutritional advice will facilitate truly personalized therapeutic recommendations. 

Another aspect explored in this Special Issue includes new and improved equipment design aiding the advancement of precision medicine. In particular, Manolesou et al. [8] describe the development and preclinical assessment of a novel bidirectional needle for aortic surgery. Aortic aneurysm repair by hand-sewn anastomosis is technically challenging, and several factors, including the stability of the surgeon’s hand and posture, as well as the needle angle and entry/exit pathway curve, can affect the outcome of this surgical procedure. The proposed bidirectional needle design resulted in shorter anastomosis completion times and improved surgical precision. Factors such as enhanced needle durability and tissue penetration also contribute to its effectiveness [8]. The future application of such tools in the clinical setting represents yet another path toward fulfilling the promise of precision medicine. 

## 4. Summary

In summary, this Special Issue covers a breadth of precision medicine aspects, including an array of new therapeutic targets and a variety of novel approaches aiming towards implementation of precision medicine against cardiac disease. The reported studies shed light on novel approaches towards a more personalized therapy against CVD that maximizes efficacy and minimizes detrimental side effects. It is anticipated that the continuously deeper understanding of the biology behind CVD, along with the striking and rapid technological advancements, will contribute to integrating personalized and targeted medicine in Cardiology [9,10].
